# Family planning for women with severe mental illness in rural Ethiopia: a qualitative study

**DOI:** 10.1186/s12978-021-01245-1

**Published:** 2021-09-28

**Authors:** Tigist Zerihun, Katherine Sorsdahl, Charlotte Hanlon

**Affiliations:** 1 Alan J. Flisher Centre for Public Mental Health, Department of Psychiatry & Mental Health, University of Cape Town, Cape Town, South Africa; 2 Department of Psychiatry, St Paul’s Hospital Millennium Medical College, Addis Ababa, Ethiopia; 3 Centre for Global Mental Health, Health Service and Population Research Department, Institute of Psychiatry, Psychology and Neuroscience, King’s College London, London, UK; 4 Department of Psychiatry, School of Medicine, College of Health Sciences, Addis Ababa University, Addis Ababa, Ethiopia; 5 Centre for Innovative Drug Development and Therapeutic Trials for Africa (CDT-Africa), College of Health Sciences, Addis Ababa University, Addis Ababa, Ethiopia

**Keywords:** Family planning, Psychosis, Contraception, Task-shifting, Primary health care, Global mental health, Community mental health, Community health worker

## Abstract

**Background:**

Family planning is a crucial issue for all women of reproductive age, but in women with severe mental illness (SMI), there may be particular challenges and concerns. As primary care-based mental health services are expanding in low- and middle-income countries (LMICs), there is an opportunity to improve family planning services for women with SMI. However, research exploring unmet family planning needs of women with SMI in such settings is scarce. Therefore, the present study explored the family planning experiences and preferences of women with SMI who reside in a predominantly rural area of Ethiopia.

**Methods:**

A qualitative study was conducted. Women with SMI who were participating in the ongoing population-based cohort study in Butajira were selected purposively based on their responses to a quantitative survey of current family planning utilisation. In-depth interviews were conducted with 16 women with SMI who were of reproductive age. Audio files were transcribed in Amharic, translated into English and analysed thematically.

**Results:**

Participants reported pervasive effects of SMI upon the intimate relationships and sexual lives of women. Although women with SMI felt that family planning was important, they had limited knowledge of family planning generally, and a lack of understanding of the specific family planning needs relevant to having SMI. None of the women with SMI in the present study had received any recommendations to use family planning services while accessing mental health care services. The participants identified ways in which primary care-based mental health services could better meet their family planning needs.

**Conclusions:**

This study provided in-depth perspectives from women with SMI about the broader context of their family planning experience, needs, barriers and how integrated primary care services could better meet their needs. Empowerment of women with SMI to access information and services needs to be an important focus of future efforts to improve the reproductive experiences of this vulnerable group.

**Supplementary Information:**

The online version contains supplementary material available at 10.1186/s12978-021-01245-1.

## Plain language summary

Family planning is a crucial issue for all women of reproductive age, but in women with severe mental illnesses (SMI), including conditions like schizophrenia, bipolar disorder and severe depression, there may be particular challenges and concerns. Very little is known about the unmet family planning needs of women with SMI who live in low- and middle-income countries. In this study, we explored the family planning experiences, unmet needs and preferences of women with SMI who live in a rural area of Ethiopia. We identified women with SMI who were taking part in a community study asking about their use of family planning. We selected 16 women with low and high use of family planning to interview in depth. The study women spoke of how having a mental illness drastically affected their intimate relationships and sexual life. Although the women felt that family planning was important, they had limited knowledge of family planning generally and a lack of understanding of the specific issues that mental illness might have on family planning choices. None of the women had received any recommendations to use family planning services while accessing mental health care services. The participants identified ways in which primary care-based mental health services could better meet their family planning needs. We concluded that it is important to focus on empowerment of women with SMI in this rural Ethiopian community, making sure that they have access to the kind of information and services that they need for family planning.

**Supplementary Information:**

The online version contains supplementary material available at 10.1186/s12978-021-01245-1.

## Background

A high priority has been given to expanding access to family planning in low- and middle-income countries (LMICs). However, there is concern that women with mental health conditions may not have benefited equally from these initiatives. There are significant health and socioeconomic advantages when women can access family planning and exercise the right to use or not to use contraceptives [1, 2]. Although family planning is an important issue for all women of reproductive age, there are a number of reasons why it may be more complex and challenging for women living with severe mental illness (SMI).

One challenge is that women with SMI have higher rates of unplanned pregnancy than the general population [3]. Marengo and colleagues found that 37.7% of pregnancies were unplanned among women with SMI, while only 9.6% were unplanned in women without mental illness [4]. In another study, women with schizophrenia were twice as likely to have unplanned and unwanted pregnancies than healthy controls [5]. A host of reasons may be associated with less effective family planning in women with SMI. Medication interactions between some psychotropic medication and hormonal contraceptives may render contraception less effective [6]. Symptoms of mental illness, such as sexual disinhibition in mania, can directly increase the risk of unplanned pregnancy or may interfere with a woman's capacity to access or utilise family planning [7]. Severe mental illness also increases the vulnerability of women and may undermine their ability to negotiate the use of various family planning methods [8–10].

Women with SMI also have increased risks of pregnancy, birth and neonatal complications. This includes risk of placental abnormalities, ante-partum haemorrhage, preterm labour and fetal distress, with the infants more likely to be small for gestational age, of low birth weight and at higher risk of sudden infant death syndrome [11–14]. SMI is a risk factor for poor obstetric outcome, including perinatal mortality (estimated 2.4-fold increased odds) and congenital malformations (estimated 1.4-fold increased odds) [15]. Some of these complications may be directly related to SMI [16, 17]. However, even after adjusting for socioeconomic status, women with SMI tend to have poorer health than their counterparts without SMI due to mental illness treatment and lifestyle factors such as smoking and lack of exercise [11, 12].

Family planning is particularly important for women with SMI due to the perinatal effects of psychotropic medication. The risk of teratogenicity from some psychotropic medications is highest during the first trimester of pregnancy, with the critical period of exposure often occurring before pregnancy is detected or disclosed [18]. Moreover, discontinuation of the medication during pregnancy increases the risk of relapse [11]. Psychotropic medications may also cross into breast milk, which adds an important consideration in settings with high levels of exclusive breast feeding and no realistic option to bottle feed [19–21].

Taking the challenges and complexities faced by women with SMI in relation to pregnancy and childbearing, family planning and education about future pregnancy is of critical importance in a woman living with an SMI during childbearing years. It is recommended that information about family planning should be incorporated into regular health care services [5]. However, access to family planning advice and methods is low in most LMICs and is likely to be even lower in women with SMI, thereby limiting the effectiveness of this approach. For example, sub-Saharan Africa has low levels of family planning coverage, estimated to be 18.4% [22]. Even though family planning constitutes a central element of primary care in Nigeria, there is no family planning service targeted at women with SMI [23]. To date, very few studies have investigated the issue of family planning among women with SMI [24]. In a recent systematic review on family planning amongst women with SMI, the studies were predominantly conducted in tertiary healthcare settings and high-income countries [23].

In Ethiopia, family planning coverage is estimated to be 41% among married women and 45% in the Southern Nations, Nationalities and Peoples’ region of Ethiopia, with lower utilisation in unmarried women. Poorer access to family planning is associated with lower education, lower socioeconomic status and living in a rural area [25]. Depression and anxiety symptoms have been associated with greater unmet needs for family planning in rural Ethiopia [26]. Although Ethiopia has rigorous evidence on the prevalence of SMI from community studies (1.3%; 37.8% women), the impact of SMI on family planning has not been investigated in a comparative study [27]. In a study of women attending psychiatric outpatient care in Addis Ababa, Ethiopia's capital, only 68% of women with SMI were able to mention one family planning method and 38% of women expressed a low intention to use family planning in the future [28]. This is substantially lower than findings from a community study in the general population where 100% of women were aware of family planning methods, and 88% expressed favourable attitudes to using family planning [29]*.*

At present, no studies have explored reasons for low access to family planning in women with SMI in Ethiopia. There is also no published evidence on how women with SMI prefer family planning services to be rendered. The plans to scale up mental health care by integrating into primary care services in Ethiopia [30] provide a potential opportunity to deliver more holistic care to women with SMI and improve their access to family planning advice.

## Methods

### Objectives

1. To investigate the family planning experiences of women living with severe mental illness.
2. To explore the attitudes of women with severe mental illness towards family planning interventions and their unmet needs.

#### Study setting

This study was conducted in and around Butajira, a town located in the Southern Nations, Nationalities and Peoples' Region (SNNPR) of Ethiopia. The first language of most of the population is a dialect of Guragigna, but Amharic is widely spoken, and there is substantial interaction among communities.

#### Study design and sample

A qualitative study design was nested in the ongoing Butajira SMI cohort study, in which participants enrolled based on the Statistical Manual of Mental Disorder 4th edition (DSM-IV TR) [31]. Women of childbearing age (18 to 49 years) with an SMI Diagnostic and Statistical Manual of Mental Disorders 4th edition (DSM-IV) diagnosis of schizophrenia, bipolar disorder or severe major depression) were invited to participate in the study. Out of the 68,378 people initially screened during the establishment of the cohort between 1998 and 2001, a total of 919 people were found to have SMI. Of these, 347 were women in their reproductive age at inclusion in the cohort [32]. During this study period (2015/16), only 188 women remained in the reproductive age category, of whom 113 participated in an initial quantitative survey about family planning practices and preferences (See Additional file 1). Non-participation in this survey was due to amenorrhea for more than two years or reported menopause (N = 24), not located at home after two visits (n = 16), permanent address change (n = 5), vagrant or living on street (n = 4), death (n = 3), hearing difficulty (n = 2) and unwillingness or refusal to consent (n = 26). The family planning survey respondents provided a sampling frame from which we could purposively select women to participate in this qualitative study.

#### Sampling and interview procedure

We purposively selected participants based on: (1) their current family planning practices and preferences; (2) educational level, and (3) diagnosis. Although eight interviews were anticipated, more interviews were required to reach theoretical saturation. Participants were informed of the nature and aim of the research, and their capacity to give consent was assessed. Participants with the capacity to consent were informed about the research and invited to provide written consent. The information sheet was read to the participant by the first author. Non-literate women signified consent with a fingerprint while another literate person witnessed that the information sheet was fully described to the participant by the researcher. All in-depth interviews were conducted in Amharic, the official language of Ethiopia. The first author (TZ), who is a psychiatrist trained in qualitative data collection, conducted all in-depth interviews. The interviews were conducted at Butajira Hospital Psychiatric Unit, with privacy and confidentiality assured at all times. An interview guide covering the areas of interest of this study was prepared, pretested in a different setting (Additional file 2).

In terms of positionality, the interviewer was a female psychiatrist from Addis Ababa and was aware that respondents might not feel comfortable disclosing their perspectives on family planning if they ran counter to a biomedical narrative. Furthermore, it was possible that non-literate, rural women might feel that the interviewer was an 'expert' due to her educational level. The interviewer sought to mitigate these effects by developing a warm rapport with participants, emphasising that she was interested to understand their perspective and that there were no right or wrong answers.

The interviews lasted between 35 min and an hour. All interviews were audio-recorded, with the permission of the participants. Transportation cost was covered for participants and their attendants to come to the Hospital. Ethical approval was obtained from the University of Cape Town's Human Research Ethics Committee and from Amanuel Mental Specialized Hospital's research ethics review board.

#### Data analysis

The audio-recorded interviews were transcribed verbatim by experienced transcribers. Amharic transcriptions were checked against the audio files and the field notes taken by the psychiatrist (TZ) before being translated to English for analysis by psychiatric residents and psychiatrists. The qualitative data analysis software, Open Code version 4.02 was used to facilitate systematic data management and analysis. We used thematic analysis, using a mostly inductive approach to capture emerging themes.

Initially, interview transcripts were read for emergent themes, which were then coded. Care was taken to ensure that the codes accurately captured the participant's ideas. Another mental health professional coded three transcripts independently, coding schemes were compared and disagreements were discussed, and consensus reached with the second author (CH). The first author and another psychiatrist coded all the remaining transcripts, applying the identified codes and drawing upon additional codes where the data required, frequently discussing with the other two authors to ensure the validity of the categories. Themes were developed from the codes. Illustrative quotes were selected for each theme. During the analysis, we considered the influence that interviewer positionality might have had on responses and focused on elaborated responses and personal examples provided by the respondents. The study has been reported in line with the Consolidated criteria for reporting qualitative studies COREQ) (See Additional file 4).

## Results

The findings of the study are presented according to the following four major themes that emerged from the data analysis: (1) the broader context of intimate relationships and sexual life of women with SMI; (2) attitudes towards childbearing in women with SMI; (3) experience of family planning in women with SMI; and (4) preferred family planning services. Detailed information about themes, major codes and sample quotes are provided in Additional file 3.

### Participant characteristics

In-depth interviews were conducted with 16 participants. The age of the participants ranged from 23 to 40 years old. More than half of the participants were unable to read or write (n = 9). Most participants described themselves as housewives (n = 11) or unemployed (n = 4), with the remainder reporting craftwork and petty trade activities. The majority of participants were single (n = 10). The diagnoses of the participants, as provided by the Butajira cohort study were schizophrenia (n = 4), bipolar disorder (n = 6) and severe major depression (n = 6) (see Table 1).

**Table 1:** Descriptive characteristics of participants (n = 16)

Characteristics	Frequency	Percent
Age (mean, standard deviation)	35.3 (6.23)	
Diagnosis		
Schizophrenia	4	25.0
Bipolar disorder	6	37.5
Major depressive disorder	6	37.5
Marital status		
Single	7	43.8
Married	6	37.5
Separated	2	12.5
Divorced	1	6.3
Residency		
Rural	11	68.8
Urban	5	31.3
Education		
No education	9	56.3
Informal	2	12.5
Formal	5	31.3
Occupation		
Housewives	11	68.8
Self employed	2	12.4
Unemployed	3	18.8
Taking psychotropic medication		
No	7	43.3
Yes	9	56.3

### Context of intimate relationships and sexual life of women with SMI

Many of the participants perceived that mental illness had impacted upon their personal relationships. They felt that people in the community did not consider a woman with SMI to be a person fit for friendships, intimate relationships and sexual life. They reported that women with a mental illness were defined and sstigmatised by their illness, with this being considered to be the only thing worth their focus in life. Not only was this prejudice held by community members, but also shared by some of the health care professionals. Many of the women themselves endorsed the view that their mental illness should be the only concern in their life. Such attitudes led to a disruption of their relationships, if they had any. Many of the women spoke of such experiences:

They [people] think that a mentally ill woman doesn't have extra needs beyond thinking about her illness.

Single woman with schizophrenia (ID07)

Since she is mentally ill, she is considered as good for nothing and not able to get a man to marry her.

Single woman with bipolar disorder (ID14)

A number of participants reported that women living with an SMI were highly vulnerable to abuse and sexual assault as a consequence of their illness. Others added how mental illness compromised women's ability to assert their rights, sometimes being forced to engage in behavior with which they were uncomfortable. A number of participants reported that they had been the victim of sexual violence or assault. Respondents described situations where men coerced women with SMI into engaging in various sexual acts:

… I have a small cottage, and I have a small piece of land. I do my own work while I was living like this until one day, he forced himself on to me. I didn't like him, I didn't will it, he didn't talk to me.

Single woman with schizophrenia (ID02).

When I was sick, someone who was living in our village deceived me. He told me that he would take me and marry me. He is a friend of my brother. Then he played tricks on me. Then, when my brother intimidated him, he stopped his action… When I got angry at home, I did something… I went out from home. It was at night and he forced [raped] me; he knows that I am mentally ill.

Single woman with bipolar disorder (ID12)

Furthermore, for many of these women sexual assaults brought them unwanted pregnancies. The woman who had been assaulted by her half-brother spoke graphically of the assault as being like death for her:

I have a child from my brother…. It is embarrassing when your brother killed you and he lives his comfortable life. He went abroad a few days after he buried me [forced me].

Single woman with bipolar disorder (ID16)

Many participants observed that often people would comment negatively when a woman with SMI gave birth to a child after being sexually assaulted. The participants perceived that the community was judgmental towards women with SMI in this situation. For example, a homeless woman with SMI who had a child following a sexual assault was seen in the same way as a healthy woman who had a child out of wedlock. This is what a woman who had given birth recounted of what people had said to her:

They say "You are mentally ill and you give birth to a bastard?" and she replies "I am on the line [I am homeless]. What can I do?” They say "How can she give birth while she has mental illness?"

Married woman with bipolar disorder (ID06).

Other women also described how their intimate relationships had been negatively affected by mental illness. When their partners discovered that they had a mental illness or they witnessed a relapse, their relationships came to an end, either by separation or divorce. Whether this was a formal or informal relationship, the outcome was usually the same:

He left me alone. He didn't say a word, he left town, and he hid after he knew I gave birth and…. Umm that occurred to me when he knows I am mentally ill.

Single woman with schizophrenia (ID02).

### Childbearing in women with SMI

Participants had various concerns about child bearing in women with SMI, such as a fear of relapse of the illness during birth or after delivery, being unable to raise their children and difficulty in parenting, and the effect of the medication on their child. For all these reasons, most (n = 11) participants reported that women with an SMI should not give birth. The most emphasised reason for a woman with SMI not to give birth was the risk of relapse. Participants tended to attribute the relapse of the mental illness in the post-partum period solely to the existing mental illness. Participants did not mention the role and impact of psychological and social factors. One participant shared her experience of illness relapse in relation to childbirth:

… When I was still having children, I used to suffer from a mental illness. It relapsed when I delivered. I am very sick now, this year it's worse. Giving birth isn't good with my mental illness… In my opinion; the child should have not have been born. When giving birth, the mental illness starts again… Yes, I got sick. That's why I say I don't want to have children.

Married woman with major depressive disorder (ID09)

Aside from relapse, the second other major concern about childbearing was about not being able to care for their newborn baby. A number of participants reported that they were not able to provide adequate care, for example, with housekeeping and cooking, when their family support was either inadequate or non-existent. However, these worries were focused predominantly on the physical needs of the newborn. Emotional aspects of parenting were not mentioned by the study participants:

…. Yes, it's hard, it's even harder to manage ourselves let alone a child……A child cannot take care of himself. He can't keep himself clean or he can't even feed himself.

Single woman with schizophrenia (ID07)

Only one participant expressed fears that she might give birth to a child with health problems because of exposure to medication taken for the mental illness. Despite her concerns, this woman had never raised this issue for discussion with a health professional or others, and no one had given her any information about this issue:

She [a woman with mental illness] is on psychiatric medication and if she gets pregnant and gives birth, what is going to happen to the newborn, is he going to be mentally retarded or normal? I only ask myself about this, I never ask or talk with the health workers or with others.

Single woman with bipolar disorder (ID15)

All the participants reported that most family members, the community and some health professionals were of the view that a woman with mental illness should not have children. This was perceived by the women as a negative attitude and not only advice given for the sake of their health. One participant highlighted the paradox of being condemned for getting pregnant on the one hand, but on the other hand, having little control over whether or not she becomes pregnant as follows:

……. How can she get pregnant if the illness doesn't disappear? God's work… People talk, saying why she didn't get contraceptive injections and why she wanted to have children since she is ill… but pregnancy can come against her will by force… all people say no giving birth if she is mentally sick.

Married woman with bipolar disorder (ID01)

### Family planning experiences and awareness in women with SMI

From most of the participants, there was initial resistance to talk about their knowledge of family planning which appeared to be related to the sensitivity of the topic. Most of them equated family planning with prevention of birth, rather than planned birth, and referred only to contraceptive interventions. Injection, pills and condoms were the contraceptives which were widely recognised by the participants. Only a few of the participants expressed awareness about implants and intra-uterine contraceptive devices. None of the participants had ever heard about emergency contraceptives. Some women expressed the view that the concept of family planning refers only to limiting the number of children an individual has but does not include controlling the timing of pregnancy.

Misconceptions about family planning were evident. A majority of women considered contraception to be the only role of family planning. Some of the participants considered the definition of family planning to be only caring for the family and managing household activities**:**

… They say it is managing your home properly, caring for the family keep your hygiene, don't sleep wearing clothes, sleep just wearing night clothes, care for your children.

Single women with bipolar disorder (ID11)

Two participants considered family planning to be specifically important for commercial sex workers. The participant expressed her understanding as follows:

I think it [Family Planning] is a business. …. Business is going to males to get money…. Women who do that, they know well about it because they are afraid to get pregnant.

Single woman with bipolar disorder (ID12)

Although condom use was generally recognised as a means of contraception, condom use was more often linked to promiscuity and preventing transmission of sexually transmitted diseases rather than an intervention used in family planning. The belief in the negative associations of condom use is conveyed by the following statement:

Condom means… indecent people use condoms; these people use them to create temporary relationships… To protect themselves from different problems, when they are in temporary relation. They are ill-mannered. They used it in hotels….

Single woman with bipolar disorder (ID15)

The majority of participants expressed inconsistent knowledge about contraceptives and some of them displayed concerns and apprehension about side effects. Despite this they reported that contraception was important in preventing pregnancy and expressed a positive attitude towards its use:

For me, a woman living with mental illness shall use implant earlier or, if she wants to have sex, she shall use pills or injection so that she can prevent extra mental health complications associated with such issues.

Single woman with schizophrenia (ID16)

No woman spoke of being forced to use contraception because they were mentally ill. The main issue was that they were not able to obtain family planning services when they needed them. They reported that this was because nobody made an effort to give it to them and they experienced insurmountable barriers to accessing family planning in primary health care.

### Preferred family planning in women with SMI

Most of the study participants discussed that family planning services should be accessible for all women living with a mental illness. They spoke about the need for accessibility and privacy, and raised concerns about stigma, lack of adequate knowledge about family planning, and the need for special considerations in the family planning service. Most participants preferred to be provided with family planning services in a mental health clinic and by a mental health professional. The reason given was the need for the person advising on family planning to have adequate knowledge about mental health: The participants discussed this as follows:

We [women with SMI] need extra support, like advising and teaching slowly, as we don't have faster functioning in understanding lessons/things. But I still insist it is good if mental health physicians could teach us so persistently and with utmost perseverance.

Single woman with bipolar disorder (ID10)

Although almost all the participants preferred to receive the service integrated with their mental health care, a few suggested their home as another alternative service area for family planning in women with SMI. The reason for this being that it would reduce the distance they would be required to travel and it would ensure privacy and confidentiality.

Health extension workers should teach us and our family. … They [women with SMI] need to get frequent advice and teaching… Yes, education is good. For a mentally ill women family planning would be good when they give time just like you have given me now and when they ask us and when they help us to understand, until now nobody has done this, this is my first time.

Single woman with bipolar disorder (ID10)

Most of the participants also recommended that family planning services be offered individually and not in a group format. Most had seen family planning services provided in a group format and expressed that they would be afraid to ask questions and may find it difficult to understand the discussion as well as other group participants. Finally, participants emphasised that women with SMI need awareness about, and access to, emergency family planning services. Most of the participants expressed their interest in emergency family planning service as they are a vulnerable group.

## Discussion

This study resulted in a number of important findings, including pervasive effects of SMI upon intimate relationships and the sexual life of women, misconceptions about family planning among women with SMI and the lack of available family planning services.

To begin with, the participants in this study perceived a reduced chance of having a lifetime partner due to stigma and discrimination. These women perceived stigmatising attitudes from the community [33, 34] and endorsed some of the negative stereotypes [35]. These findings are consistent with studies on women with SMI from other parts of the world. For instance, a study from India found that community members held negative attitudes about men marrying a woman with an SMI [36]. In a study from Turkey, women with SMI held negative perceptions about marriage, sexuality, family planning, childbearing and pregnancy, compared with women without SMI in the general population [37]. As a consequence, the participants felt that they were not desirable as romantic or sexual partners. The women in this study appeared to harbour negative attitudes about their own sexual life, thereby limiting their quest for partnership options.

The study participants also noted that women with SMI tend to run much higher risks of victimisation and/or sexual exploitation. Some of the respondents spoke of their experiences of sexual assault, either at the hands of extended family members or strangers. Research indicates that women with SMI have a greater risk of victimisation than women without mental illness [38–41]. They have much higher risks of sexual abuse and posttraumatic experiences, which in turn can aggravate the mental illness [8, 42, 43]. Exacerbations of mental illness could limit women's power and ability to control their own sexual life [44–46], which further increases the risks of unplanned pregnancy.

Although women with SMI in Ethiopia felt that family planning was necessary, they had limited knowledge of family planning generally and a lack of understanding of the specific family planning needs relevant to having SMI. The most common reason for using family planning for women in the present study was for preventing pregnancy rather than birth spacing. This finding is similar to a study conducted in Nigeria where women with SMI reported that pregnancy prevention is the goal, even though the timing of having a baby was the most important problem faced by women in rural communities [23]. Women's lack of adequate knowledge about family planning and fear of side effects played a role in the decision to use contraceptives. This misunderstanding has implications for the uptake and discontinuation of contraception and accords with previous reports from Ethiopia [25, 47–50]. In the present study, a majority of the women were aware of the existence of injectables, pills and implanted contraception. Although some of the women knew about the intrauterine contraceptive device and condoms, some related condom use with promiscuity rather than a method used in family planning. This can be partly explained by the paradoxical influence of the media in advocating condoms for the prevention of sexually transmitted diseases and the cultural taboo to the disclosure of condom use [51, 52].

In terms of family planning needs specifically for women living with SMI, the participants did express fears about relapse in the context of childbearing. As evidenced by many studies, delivery appears to be one of the factors most likely to increase the risk of relapse in women with SMI [53–57]. This perception, in turn, affected the community's view of childbearing as risky for such women. Contrary to the evidence that discontinuing medication during pregnancy opens up the possibility for relapse in women with mental illness [58–60], participants did not mention the risk of relapse associated with drug discontinuation or special treatment needs during pregnancy. Instead, women emphasised the stresses associated with childbearing, such as inadequate sleep, nutrition and support. However, although the women in this study had access to psychiatric nurse-led outpatient care, this centralised service was unlikely to meet the needs of perinatal women with SMI adequately. Expectations of even specialist mental health services in Ethiopia have been found previously to be low [30, 61, 62], and this may be a barrier to improving care for perinatal women with SMI.

Finally, even though women of reproductive age with an SMI are vulnerable to unplanned pregnancy, are at risk for mental health relapse during childbearing and could be exposed to psychological and economic burdens if an unplanned pregnancy occurs, none of the women with SMI in the present study had received any recommendations to use family planning services while receiving mental health care services. Integrating family planning services into specialist mental health services was generally preferred by the women in this study, in keeping with the first choice of women with SMI in high-income countries [50]. Participants emphasised the need for specialist knowledge, for example, about interactions between their medications with contraceptives, the illness and the family planning approach. They also felt more comfortable communicating with mental health professionals, with whom they had built up a relationship over a long period of time. The women in our study reported that there was a need for improved family planning advice and referral from mental health professionals. This result supports previous studies highlighting that recommendations by health professionals regarding family planning recommendation need to be modified to address specifically the needs of women with SMI [63, 64].

Family planning services in Ethiopia are usually accessed through all levels of general health care services. However, few health professionals in Ethiopia deliver a comprehensive range of family planning methods suitable for women with SMI [65]. In part, this reflects the low priority which has been given to mental health care in the training of general health care providers. The plan to expand mental health care by integrating into primary care may provide an opportunity to better meet the family planning needs of women with SMI. However, there may need to be modifications for women with SMI.

Several limitations affect the scope and breadth of the current study and the analysis of the findings. Purposive sampling was used in order to obtain in-depth information from women selected on the basis of differing family planning practices, but this means that participants may not have been representative of all women with SMI. Another potential limitation was that the sample was recruited from a cohort study in which the participants had relatively better access to mental health services and treatment. Therefore, the results of the present study are not representative of all women with SMI in the country and the findings may be difficult to generalise to other contexts in Africa. However, the Butajira cohort was community-ascertained and not liable to the strong selection bias seen in facility-based studies in this context. The findings of extremely low levels of awareness and problems of access, even in a relatively better-served population, indicate that the study is likely to be of value for service development in other areas of rural Ethiopia. The other possible limitation of this study could be that its scope includes only women with SMI. The study excluded other groups of participants who are involved in the care of women with SMI. This includes health extension workers, primary health care professionals, psychiatric nurses, their caregivers and community representatives. Future research needs to look at the views and experiences of the other stakeholders, such as health extension workers. Including these participants and having their perspectives may contribute to the development of a feasible and acceptable intervention.

## Conclusions

This study is the first of its kind in Ethiopia to explore family planning among women with SMI in a rural low-income country setting. Current family planning experiences were not in keeping with women's preferences in relation to the venues or providers of the service. Furthermore, they could not access the information they needed. Stigmatising attitudes towards women with SMI having relationships and a sexual life constrained access. Women's vulnerability to exploitation and exclusion was also evident. We concluded that it is crucial to focus on the empowerment of women with SMI in this rural Ethiopian community, equipping them to exercise their rights to express preferences and access the kind of information and services they need for family planning.

Future studies need to focus on developing and evaluating contextually relevant interventions to support FP access for women with SMI. These need to be provided in the context of sexual and reproductive health more generally, addressing structural barriers to their exclusion from care and focusing on women's empowerment to equip them to exercise their rights to information and obtain their preferred FP interventions. Broader community-level interventions are also needed to address the stigmatisation and exploitation of women with SMI.

## Supplementary Information

**Additional file 1.** Questionnaire for quantitative interview for women with SMI.

**Additional file 2.** Interview guide for women with severe mental illness.

**Additional file 3.** Themes, major codes and sample quotes.

**Additional file 4.** Consolidated criteria for reporting qualitative studies (COREQ): 32-item checklist.

## Data Availability

The data that support the findings of this study are available. The data are not publicly available due to their containing sensitive information that could compromise the privacy of the participants.

## Abbreviations

DSM- IVTR: Diagnostic and Statistical Manual of Mental Disorders, 4th edition text revision.
FMOH: Federal Ministry of Health
LMICs: Low and Middle-Income Countries
SMI: Severe Mental Illness
WHO: World Health Organization
