# Supplementary material for: Family planning for women with severe mental illness in rural Ethiopia: a qualitative study
Source: Reprod Health. 2021 Sep 28;18:191. doi: 10.1186/s12978-021-01245-1 (PMC8480012; doi:10.1186/s12978-021-01245-1)
Supplement: Supplementary file 1 — Additional file 1. Questionnaire for quantitative interview for women with SMI. [file 12978_2021_1245_MOESM1_ESM.docx]

Additional file 1 : - Questionnaire for quantitative interview for women with SMI

Please answer the questions below

Key Demographics

1. Age ---------------------------------------
2. Marital status -------------------------
3. Employment --------------------------
4. Educational level ----------------------------
5. Place of residence 1) rural 2) urban
6. Diagnosis ----------------------------------
7. Are you taking medication? 1) yes 2) No
8. If yes, when did you start taking medication? ----------------------------
9. List current medication and dose:
10. Do you have children? 1) yes 2) No
11. If yes, how many children do you have? ----------------------
12. Are you pregnant currently? 1 = Yes 2 = No [If no, skip to question 14]
13. At the time, you became pregnant, where you using contraception?
    1. Not using contraception 2) Tablets 3) Injection 4) Natural (timing) 5) Condoms
14. Is there any method you prefer from the above methods and Why?
15. Do you want to be pregnant now? 1=Yes 2=No
16. Do you want to have more children? 1=Yes 2=No
17. If 1, do you know of any ways to prevent pregnancy? 1=yes 2=No

18. Are you using any family planning methods currently?

1) Not using contraception 2) Tablets 3) Injection 4) Natural (timing) 5) Condoms 6) sterilised (woman) 7) sterilised (partner) 8) other

1. If not using family planning methods, what is the reason?
   1. I want to have another child
   2. My husband wants another child
   3. My husband does not allow me
   4. My religion does not permit me
   5. For health reasons (either can't tolerate side effects or has medical contraindication)
   6. FP services not available
   7. I don't know about methods of preventing pregnancy
   8. Leave it to nature
   9. Don't live with husband
   10. Other, specify:
2. If using family planning methods, which do you prefer, and why?
3. Do you want to have more children in the future? 1= Yes 2 =No
