# Supplementary material for: Family planning for women with severe mental illness in rural Ethiopia: a qualitative study
Source: Reprod Health. 2021 Sep 28;18:191. doi: 10.1186/s12978-021-01245-1 (PMC8480012; doi:10.1186/s12978-021-01245-1)
Supplement: Supplementary file 2 — Additional file 2. Interview guide for women with severe mental illness. [file 12978_2021_1245_MOESM2_ESM.docx]

## Additional file 2: - Interview guide for women with severe mental illness

The main aim of the study is to explore the experiences of women living with severe mental illness of family planning interventions in Ethiopia. During this interview, I would like to get your views on the ways in which you are getting information regarding family planning, as well as your attitudes and experience related to family planning. I value each one of your thoughts and opinions. Please remember that there are no right or wrong answers. Please feel free to discuss what you think. If there are issues that you really do not want to discuss you are free to not do so. Everything that is discussed in the interview is considered confidential this interview should last for about 45 minutes to 1 hour. I would like to record the interviews with your permission, to write down every word from the recordings and then try to understand what you and the other participants have said. These responses will be presented in reports. No identifying information such as names will be included in the reports.

**Thank you for helping me with this study.**

Participant No/Pseudonym -------------------------

Date of Interview ------------------------

Interview Start time ------------------------.

Interview end time -------------------

1. Tell me a little bit more about your living conditions?

---------------------------------------------------------------------------------------------------------------------------------------------------------------------------------------------------Notes:

Prompt

*a) With whom you are living?*

*b) Who supports you? Who do you support?*

2. Please tell me what you know about family planning?

Notes: - ---------------------------------------------------------------------------------------------

Prompt: -

1. What are the different methods that you know about? Have you used any of these in the past?
2. [If relevant] What is the method of choice for you and why do you prefer that method?

3. Please tell me about your views around having children

------------------------------------------------------------------------------------------------------------------------------------------------------------------------------------------------------------------------------------Notes:- -------------------------------------------------------------------------------

Prompt: -

1. *What are your views about you having children as mentally ill women?*
2. *When should women with severe mental illness have children?*
3. *What are the ideal conditions to have children being mentally ill? (Disease condition and treatment)*
4. *See the comments what do others in your family think? Why do you think they think that way?*
5. *Are there any issues you think about regarding having children and living with a mental illness?*
6. *What are your views on How other people (E.g. health workers, relatives, communities) feel about mentally ill women bearing children having?*
7. *What do you think about the views of the community/t peoples?*

4. What do you know about current services regarding family planning in the health facility?

---------------------------------------------------------------------------------------------------------------------------------------------------------------------------------------------------------Notes: - ----------------------------------------------------------------------------------------

*Prompts: -*

1. What would you hope the Family planning service in the health facility would provide for you*?*
2. What have you actually experienced *regarding family planning service in the health facility?*
3. *How important do you think it is to have an intervention to plan pregnancy?*
4. *What extra support of information you should be given about medication, risk of unplanned pregnancy?*
5. *Tell me good things about the service you received?*
6. *Tell me any problems you face during service utilization?*
7. *Do you have any ideas for how these difficulties might be overcome?*

4. Now I would like to ask you to talk about your experiences with family planning services at the health facility, what do you think about it?

---------------------------------------------------------------------------------------------------------------------------------------------------------------------------------------------------------Notes: - ----------------------------------------------------

Prompts: -

1. *Drawing on your own experience, can you give me an example of a time when you*

*were forced with health professional or other peoples to use the family planning methods*

1. *What would be positive about having family planning Service?*
2. *What would be negative about havening family panning service? (Attitude)*
3. *What will be the barriers to have the service you mentioned?*
4. *Do health providers give you information about family planning or made referral? Were the referrals taken up do you get the service as you want?*
5. *In general, do you think women who have severe mental illness need extra help to access family planning? Why is that? What sort of help?*
6. *Can you tell me about any difficulties you face getting the family planning service? Any other difficulties?*
7. *Can you tell me your ideas for how those problems could be overcome? What would help you to be able to access family planning?*
8. *How would you describe the perfect environment for you to use family planning methods?*

5. In what ways do you think that the current family planning service could be improved?

---------------------------------------------------------------------------------------------------------------------------------------------------------------------------------------------------------Notes: - --------------------------------------------------------------------------------------

*Prompts: -*

*a) What kinds of things/ interventions approaches are in currently in place?*

*b) What would be the best ways to get Family planning service for you?*

*c) Where is the best place to get the family planning service? In the facility? In your home?*

*d) What about providing information about family planning for the women visiting this service?*

*e) What kind of help do you need from health workers or health system so that you can get the family planning service that you need?*

7) Do you have anything else to share?

----------------------------------------------------------------------------------------------------Notes:

Summary of the Whole interview
