# Supplementary material for: Family planning for women with severe mental illness in rural Ethiopia: a qualitative study
Source: Reprod Health. 2021 Sep 28;18:191. doi: 10.1186/s12978-021-01245-1 (PMC8480012; doi:10.1186/s12978-021-01245-1)
Supplement: Supplementary file 3 — Additional file 3. Themes, major codes and sample quotes. [file 12978_2021_1245_MOESM3_ESM.docx]

| **Themes** | **Major codes** | **Sample quotes** | **Participant code** |
| --- | --- | --- | --- |
| 1. Context of intimate relationships and sexual life of women with SMI | Context of intimate relationships | *They [people] think that a mentally ill woman doesn’t have extra needs beyond thinking about her illness.* | *Single woman with schizophrenia (ID07)* |
|  |  | *Since she is mentally ill, she is considered as good for nothing and not able to get a man, to marry her* | Single woman with bipolar disorder (ID14) |
|  |  | *My friends ignored me… because I have a mental illness… nowadays only have one friend.* | *Single woman with bipolar disorder (ID11)* |
|  |  | *… I have a small cottage and I have a small piece of land. I do my own work while I was living like this until one day he forced himself on to me, I didn’t like him, I didn’t will it, he didn’t talk to me.* | *Single woman with schizophrenia (ID02)* |
|  |  | *He [a relative of the woman] was at his home; he raped me and then made me have a baby: he did not marry me legally. Rather, he forced me at my adolescent age… nobody was there [In the compound] …that day… I was suffering in pain When they came back home …, they made coffee for themselves; nobody remembered and asked me what happened to me.* | Single woman with bipolar disorder (ID11) |
|  |  | *I asked my cousin to go with them [to the ceremony] or send me to my mothers’ home because this boy [her half-brother] might harm me. They didn’t allow me. I stayed at home. At that time, I was on the medication which made me sleepy and I couldn’t hear anything, as if I am dead. I felt that it was in my dream. When I woke up I was soaked with blood and I was in pain.* | *Single woman with bipolar disorder (ID16)* |
|  |  | *When I was sick, someone who was living in our village deceived me. He told me that he would take me and marry me. He is a friend of my brother. Then he played tricks on me. Then, when my brother intimidated him, he stopped his action… When I got angry at home, I did something… I went out from home. It was at night and he forced [raped] me; he knows that I am mentally ill.* | *Single woman with bipolar disorder (ID12)* |
|  |  | … *yes twice[I was raped] while I was sick. It is because of that I gave birth…I gave birth without marriage… It was by accident without my consent, I was forced. But, once it happened, I decided to bring them up.... It is the same for both children* | *Single woman with bipolar disorder (ID12)* |
|  |  | *I have a child from my brother…. It is embarrassing when your brother killed you and he lives his comfortable life. He went abroad a few days after he buried me [Forced me].* | *Single woman with bipolar disorder (ID16)* |
|  |  | *He left me alone. He didn’t say a word, he left town, and he hid after he knew I gave birth and…. Umm that occurred to me when he knows I am mentally ill.* | *Single woman with schizophrenia (ID02)* |
|  |  | *When I got ill, things did not go as he [my husband] had said. He left [the country]*  *…My illness relapsed. The man said that it was such a burden that my illness relapsed… He said that he is old and that he is not willing to deal with it at his age…. I married twice; they left me because of my illness.* | *Divorced woman with bipolar disorder (ID04)* |
| **2. childbearing in women with SMI;** | Relapse and giving birth | *… When I was still having children, I used to suffer from my illness… it is better if she [woman with mental illness] doesn’t give birth… I was suffering because of repeatedly giving birth. I was sick after almost all my deliveries…When I got pregnant and when I gave birth, my illness got worse…It is not only giving birth. Once I had a spontaneous abortion and my mind got very sick… (Mother of Eight)* | *Married woman with major depressive disorder (ID01)* |
|  | *Attitude of others about child bearing* | *They say “You are mentally ill and you give birth to a bastard?” and she replies “I am on the line [I am homeless]. What can I do?” They say “How can she give birth while* | *Married woman with bipolar disorder (ID06)* |
|  | *Attitude of others about child bearing* | *They insulted me in front of me …. How she can give birth being mentally ill?* | *Single woman with bipolar disorder* (ID11) |
|  | Relapse and giving birth | *I have a mental illness. It relapsed when I delivered. I am very sick now, this year it’s worse. Giving birth isn’t good with my mental illness… In my opinion; the child should have not have been born. When giving birth, the mental illness starts again… Yes, I got sick. That’s why I say I don’t want to have children.* | *Married woman with major depressive disorder (ID09)* |
|  | Parenting /child bearing | *When the children irritate me or when they disturb me, it just doesn’t feel right. I become sick. …I just feel anxious… And whenever I am breast feeding, I feel so unwell* | *Married woman with Bipolar disorder (ID06)* |
|  | Parenting /Child bearing | *Yes, it’s hard, it’s even harder to manage ourselves let alone a child……A child cannot take care of himself. He can’t keep himself clean or he can’t even feed himself.* | *Single woman with schizophrenia (ID07)* |
|  | Parenting/Child bearing | *it’s difficult having kids while being mentally ill…...a child can’t take care of himself, can’t cook…. how she can do that if she is sick?* | *Married woman with major depressive disorder (ID* 05) |
|  | Parenting /Childbearing | *Raising children and mental illness are troublesome… Raising children is as troublesome as the illness. I prefer she would not give birth…… Yes, raising children is as stressful as the mental illness for women an SMI…. So, I would advise they don’t give birth at all…. giving birth…. It is adding another stress on top of the mental illness.* | *Married woman with bipolar disorder* (ID06) |
|  | Parenting /childbearing | *It is a dual burden for a woman living with the illness: illness and childbirth* | *Single woman with bipolar disorder (ID14)* |
|  | Parenting /childbearing | *First of all; they [children] would have driven you crazy. When their children give them a hard time, they tell me I am lucky Allah did not give me any children.* | *Divorced woman with bipolar disorder (ID04)* |
|  | Social support/parenting | *She [a woman with mental illness] needs to get help from family members. … I will consider it conducive only if she has a family member who will supervise them [ child and mother ] and taken care of them.* | *Married woman with bipolar disorder* (ID06) |
|  | Social economic burden and parenting | *One with support and care, one with a comfortable life may give birth. What problem would [she] have? … Anyone with a comfortable life can raise [the child]. But what would someone with no comfort or poor health have? How would you have comfort if you do not have any health?* | *Divorced woman with Bipolar disorder* (ID04) |
|  | Attitude about child bearing | *I heard that getting married and having kids would renew your mind and give you relief. I took their advice and I got married and gave birth. Even though my first child died while he was in my womb, I gave birth to my current child. Thanks to God, I am fine now. Before I gave birth, I used to get sick and worry a lot. Although, we are poor and have very little to eat, when I spend time with my daughter, I feel better… yeah, I used to get sick a lot before. I used to talk alone. I had had a lot of stress and get disturbed easily, but now I am a lot better because I spend a lot of my time with my daughter.* | *Divorced woman with schizophrenia (ID08)* |
|  | Socioeconomic challenges/Childbearing | *I am ill and I am sick, with what money will I raise a child? I was worried and thought of killing myself when I gave birth. But Allah will ask me for my soul. Besides my father is poor and he is very old.* | *Single woman with schizophrenia* (ID02) |
|  | Socioeconomic challenges | *……Sometimes people feel sympathy for me so they give some money (five or ten birr) and sometimes I sell some lemons and buy some bread for the children with the money I get…This how I try to live daily.* | *Divorced woman with schizophrenia (ID08)* |
|  | Socioeconomic challenges | *When you have many kids, you don’t have enough for clothing or food… you don’t have enough for food…. And when the child is young you need to take care of both the child and yourself.* | *Married woman with major depressive disorder (ID05)* |
|  | Effect of medication | *She [a woman with mental illness] is on psychiatric medication and if she gets pregnant and gives birth, what is going to happen to the newborn, is he going to be mentally retarded or normal? I only ask myself about this, I never ask or talk with the health workers or with others.* | *Single woman with bipolar disorder (ID15)* |
|  | vulnerability | *How can she get pregnant if the illness doesn’t disappear? God’s work… People talk, saying why she didn’t get contraceptive injections and why she wanted to have children since she is ill… but pregnancy can come against her will by force*… *all people say no giving birth if she is mentally sick* | *Married woman with bipolar disorder (ID01)* |
|  | Attitude about childbearing | *The family carrying the ill woman, if she brings the child they will kill her, they say she shouldn’t give birth…. I say it is not necessary for her.* | *Single woman with bipolar disorder (ID*10) |
|  | Others attitude about child bearing | *…. It’s expected that people who are ill are insulted when they give birth…. They [people] say, how can she give birth unless she is better? How can this be acceptable?* | *Married woman with major depressive disorder (ID13)* |
|  | discrimination | *…local community even clearly says” a mentally ill like her! how could she give birth; how could she raise a child without even knowing what is good and bad for herself; how can she have a child? …. They openly say women with mental illness shouldn’t have children at all because they just cannot handle them. It is very difficult even to imagine!”* | *Single woman with schizophrenia (ID07)* |
| 3.Experience of family planning in women with SMI | FP awareness | *… I don’t have an idea [about family planning], and also, I am not married…* | *Single woman with bipolar disorder (ID 12)* |
|  | FP Awareness | *I have heard about it [condom] but I never had affairs. This is my first husband. I never had an affair. I have been living with him. I don’t know anybody else.* | *Married woman with bipolar disorder (ID06)* |
|  | FP Awareness | *Condom means… indecent people use condoms; these people use them to create temporary relationships… To protect themselves from different problems, when they are in temporary relation. They are ill-mannered. They used it in hotels….* | *Single woman with bipolar disorder (ID15)* |
|  | FP Awareness | *If I don't use these things [contraceptives], then I’m going to have a bastard /illegitimate child… it refers to an unwanted child without a father. But sometimes even if the father is there, he might not be supportive. … Well, it is the child with no father that you conceive with a random guy and bear it without your interest.* | *Divorced woman with schizophrenia (ID08)* |
|  | FP Awareness | *For me, a woman living with mental illness shall use implant earlier or, if she wants to have sex, she shall use pills or injection so that she can prevent extra mental health complications associated with such issues.* | *Single woman with schizophrenia (ID16)* |
|  | FP Awareness | *If I had known [about contraceptives], it would have been good for me… good for my mind… its good for my children. My condition got worse when I give birth. If I had known, it would have been good… for my sons… if they had brought it to me… I should have learnt a lot of things, my family suffered a lot when I am sick.* | *Married women with major depressive disorder (ID01)* |
|  | FP Awareness | … *They say it[Family planning] is managing your home properly, caring for the family keep your hygiene, don’t sleep wearing clothes, sleep just wearing night clothes, care for your children.* | *Single women with bipolar disorder (ID11)* |
|  | FP Awareness | *I think it [Family Planning] is a business. …. Business is going to males to get money…. Women who do that, they know well about it because they afraid to get pregnant.* | *Single woman with bipolar disorder (ID12)* |
|  | FP practice | *……. How can I take that [the contraceptive]? I am on a drug for mental illness. I can’t add more drugs. Since I am on a drug for mental illness, I told them [health professionals] I can’t take another drug. I told them I can’t. They did nothing……… They left me. They said “you may give birth if it’s God’s will, if not it’s his work” they kept quiet. They said I can’t take the drug…… Drug for mental illness is heavy; they [health professionals] said it’s difficult to take both.* | *Married woman with bipolar disorder (ID03)* |
|  | FP experience | *If I knew that [preventing unplanned pregnancy] would have been good, but I do not know, I can only think about myself, who am I to think about others? I couldn’t even do my job, I stayed at home, I couldn’t hang out with neighbouring women, I stayed home it was hard time still it is hard to me.* | *Single woman with schizophrenia (ID02)* |
|  | FP experience | *…. There are times where I took the contraceptive pills…. My mother used to take the pills, back then, I used to take her [my mother’s] pills, whenever I had unexpected sexual intercourse.* | *Divorced woman with schizophrenia (ID 08)* |
|  | FP service suggestion | *I prefer the government institutionalizes the women and take care of them. The women might not agree. But if they agree, that would be better. They can be rescued from all the compounded suffering- the mental illness and taking care of unwanted kids. … women with SMI should be institutionalized and forced into taking contraceptives. … I think it is better if [women with mental illness] are coerced into taking it. … The illness and giving birth is a struggle. So, if they don’t have a family, they should be institutionalized and given the medications.* | *Married woman with bipolar disorder (ID06)* |
|  | Discrimination in the service | *…. The other reason is because I am mentally ill, how could they [health extension workers] think about a mentally ill woman and tell her about birth control? They think that a mentally ill woman doesn’t have extra needs beyond thinking about her illness.* | *Single woman with schizophrenia (ID07)* |
|  | Perception of stigma FP service utilization | *It is embarrassing…… I have a mental illness and also, I am not married. How can women like me talk about contraceptives? It is shameful to do that* | *Divorced woman with bipolar disorder (ID04)* |
|  | Perception FP service utilization | *How could I ask for birth control when I am mentally ill?* | *Single woman with schizophrenia(ID02)* |
|  | Others attitude FP service utilization | *They say ‘why does she need it; why doesn’t she treat her illness? … They say ‘she wants to use contraception even though she has a mental illness?’* | *Single woman with Bipolar Disorder ID10)* |
| 4. preferred family planning services. | FP Attitude | *It is embarrassing…… I have a mental illness and also, I am not married. How can women like me talk about contraceptives? It is shameful to do that* | *Divorced woman with bipolar disorder (ID04)* |
|  | FP Attitude | *How could I ask for birth control when I am mentally ill* | *Single woman with schizophrenia (ID02)* |
|  | Others attitude about FP | *They say ‘why does she need it; why doesn’t she treat her illness? … They say ‘she wants to use contraception even though she has a mental illness?’* | *Single woman with Bipolar Disorder (ID10)* |
|  | FP self-Discrimination | *… If it [Family planning service] is given equally to all… yes, but it is difficult for unmarried and mentally sick woman to use the service freely.… They say, why does she need it? Why doesn’t she treat her illness?* | *Single woman with bipolar disorder (ID10)* |
|  | FP service provision methods | *Health extension workers should teach us and our family. … They [women with SMI] need to get frequent advice and teaching… Yes, education is good. For a mentally ill women family planning would be good when they give time just like you have given me now and when they ask us and when they help us to understand, until now nobody has done this, this is my first time.* | *Single woman with bipolar disorder (ID10)* |
|  | Service provider preference | *…. There has to be special advice for them [women with SMI]. … They need to have an advice by physicians here [Psychiatric clinic] that has to be provided to their [women with SMI] family and their neighbor about the need for family planning for women like me. … If there is a bad neighbor, he/she may aggravate the illness. They insult and humiliate you if you give birth or go to the birth control service. So, in such a situation, it is essential that mental health professional should also sensitize neighbors as well. …… because they are ill I would suggest, it is essential to advise and teach with great patience of these women* | *Single woman with bipolar disorder (ID14)* |
|  | Service provision method preference | *We [women with SMI] need extra support, like advising and teaching slowly, as we don’t have faster functioning in understanding lessons/things. But I still insist it is good if mental health physicians could teach us so persistently and with utmost perseverance.* | *Single woman with bipolar disorder (ID10)* |
|  | Service area preference | *I think it would be better if it is given here, when I come here [psychiatric OPD] for my follow up I could also get the family planning service… If the service is here we will get both medications here, we won’t have to go to different places* | *Single woman with schizophrenia ID02* |
|  | Service provider preference | *It’s the health extension workers who travel from house to house to give birth control [contraceptives]. It’s better for us if it was a mental health professional.* | *Married women with major depressive disorder (ID09)* |
|  |  | *I prefer if the mentally ill women could get it at home. I mean, if she could be given at home in the morning. … I choose she is given at her home. … Hmm…. I prefer it that way. … It is better that way.* | *Married woman with bipolar disorder (ID06)* |
|  | FP Service privacy | *For me, it would be good to provide counselling separately for a woman living with mental illness on such issues … the possibility of becoming a mother, … If you advise them in a group, it is difficult for them to ask questions and also, they may need additional time to understand what it has been said.* | *Married woman with major depressive disorder (ID 09)* |
|  | Service experience | *In addition, there is a privacy problem. Women say this situation makes them to be frightened. It is just like that. It is conducted in a group.* | *Single woman with bipolar disorder (ID 14)* |
|  | Service preference | *It should be in private it’s scary when it’s in groups…Yes, and when she comes in the countryside to my house I can talk to her on private… I want to talk in private and also get the medication in private. That’s how I am… yes, if they all speak to experts like you in private it’ll be good if they would give me the medication in private and also speak to me that way.* | *Married woman major depressive disorder (ID05)* |
